# Diagnosis and microsurgical treatment of chondromas and chondrosarcomas of the cranial base

**DOI:** 10.3892/ol.2014.2072

**Published:** 2014-04-16

**Authors:** SUMIN GENG, JUNTING ZHANG, LI-WEI ZHANG, ZHEN WU, GUIJUN JIA, XINRU XIAO, SHUYU HAO

**Affiliations:** Department of Neurosurgery, Beijing Tiantan Hospital of Capital Medical University, Beijing 100050, P.R. China

**Keywords:** chondroma, chondrosarcoma, cranial base, diagnosis, microsurgery

## Abstract

Chondromas and chondrosarcomas of the cranial base are rare neoplastic diseases. The aim of the present study was to evaluate the diagnosis and microsurgical treatment of these difficult cranial base tumors. A total of 19 patients who underwent microsurgery were pathologically diagnosed with cranial base chondromas or chondrosarcomas and their clinical data was reviewed. The chondromas and chondrosarcomas of the cranial base in the present study commonly originated in the sphenopetrosal, sphenoclival or petroclival junctions, and the majority were located in the parasellar region of the middle cranial base extradurally. The most frequent symptoms were headaches and cranial nerve palsy, and the Karnofsky performance score (KPS), assessed pre-operatively, averaged at 87.1. A frontotemporal or preauricular subtemporal-infratemporal approach was used in 11 cases, a tempo-occipital transtentorial or presigmoid supratentorial-infratentorial approach was employed in six further cases, and the far-lateral or retrosigmoid approach was applied in the remaining two cases. A total or near-total tumor removal was secured in 13 cases, while a subtotal removal was obtained in another five and a partial removal was achieved in one case. The most common post-operative complications included cranial nerve palsy and cerebrospinal fluid leakage, but there were no post-operative fatalities. A total of 15 patients were followed up for a mean of 67.2 months (range, 5–140 months), and 13 (76.5%) of these patients were living normal lives (KPS, 80–90). There were two patients with recurrent tumors. The neuroradiological examinations and the presenting symptoms and signs allow the pre-operative diagnosis to be presumed for the majority of cranial base chondromas or chondrosarcomas. Surgical resection is the key treatment for these tumors, and this treatment is known to improve the survival rates.

## Introduction

Chondromas and chondrosarcomas of the cranial base are infrequent neoplastic diseases, with the majority of tumors occurring in the lateral area of the cranial base on the midline. Due to the deep location and the invasion to the cranial nerves and critical vascular structures, total resection of the tumor, a difficult challenge for neurosurgeons worldwide, is extremely difficult and is frequently accompanied by various complications (1). In addition, the majority of chondromas and chondrosarcomas arise *de novo* and are common in patients with Ollier’s disease, Maffucci syndrome, Paget’s disease and osteochondroma. Several histological subtypes of chondrosarcoma have been reported, including conventional, mesenchymal, clear cell and dedifferentiated subtypes (2). Chondromas and chondrosarcomas grow slowly, but are locally aggressive and prone to relapse (3). To date, few series have analyzed chondroma and chondrosarcoma and a number of controversies remain concerning the appropriate treatment modality. The present study investigated 19 patients who were diagnosed with cranial base chondromas and chondrosarcomas and surgically treated in the Neurosurgery Department of Beijing Tiantan Hospital of Capital Medical University (Beijing, China) between 1998 and 2010. Neuroradiological examination and surgical treatment data were collected from the hospital records and reviewed. Patient symptoms, treatments and outcomes are presented.

## Patients and methods

### Patient characteristics

The study group consisted of 10 males and 9 females, with a mean age of symptom onset of 33.6 years (range, 15–57 years). The time from the onset of symptoms to the pathological diagnoses ranged from 2 months to 12 years. The presenting symptoms varied amongst the patients. In total, 12 presented with headaches and dizziness, 13 with double vision and abducens palsy, seven with poor vision and papilledema, four with facial numbness and paresis, three with tinnitus and decreased hearing, two with convulsion and two with hoarseness, decreased gag reflex and tongue atrophy. The average pre-operative KPS was 87.1 (range 60–90). Patients provided written informed consent.

### Neuroradiological examinations

**Figure 1:**
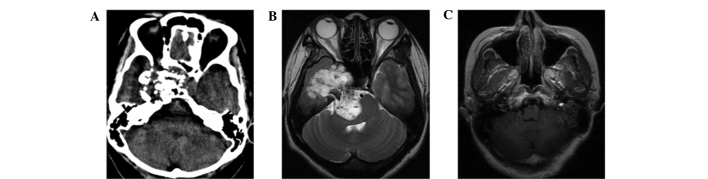
Pre-operative images showing lesions located in the parasellar region, the middle and posterior cranial fossa and the jugular foramen region, respectively. (A) Contrast-enhanced CT scan showing a mass lesion located in the right parasellar region. (B) T2-weighted MRI scan showing a mass lesion extending from the middle fossa to the posterior cranial fossa. (C) T1-weighted MRI scan with contrast showing an enhancing mass in the left jugular foramen region. CT, computed tomography; MRI, magnetic resonance imaging.

All patients underwent computed tomography (CT) and magnetic resonance imaging (MRI). Of the 19 tumors, 11 were located in the parasellar region (Fig. 1A), six extended from the middle cranial fossa to the posterior cranial fossa (Fig. 1B) and two were located in the jugular foramen region (Fig. 1C). The mean maximum diameter of the tumors, as assessed by MRI, was 4.9 cm (range, 3.1–8.3 cm). In three patients, cerebral angiography was carried out and the tumors showed no or mild staining.

### Surgical treatments

All 19 patients underwent surgical procedures. The frontotemporal or preauricular subtemporal-infratemporal approach was used for the tumors located in the parasellar region in 11 cases, the tempo-occipital transtentorial or presigmoid supratentorial-infratentorial approach was used for the lesions involving the middle cranial fossa and the posterior cranial fossa was employed in another six cases, and the far-lateral or retrosigmoid approach was applied for the lesions located in the jugular foramen region in two cases.

## Results

### Surgical findings

**Figure 2:**
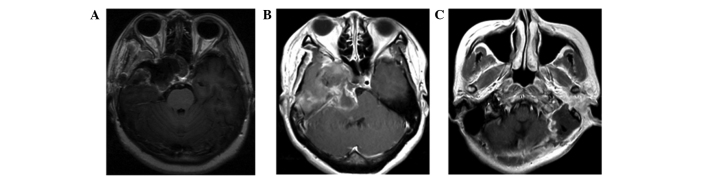
Post-operative MRI scans with contrast showing (A) total and (B and C) near-total tumor resection. MRI, magnetic resonance imaging.

Of the 19 patients, 13 underwent a total or near-total resection (Fig. 2A–C), five underwent a subtotal resection and one underwent a partial resection. Post-operatively, new cranial nerve disorders were the most frequent complications, including six cases of oculomotor nerve palsies and two disorders of cranial nerves VII and VIII. In addition, two patients suffered cerebrospinal fluid leakage, with recovery following lumbar drainage, and one patient suffered a post-operative brain infarct, with recovery following anti-ischemic treatment. There were no post-operative fatalities.

### Imaging findings

CT scans showed the tumors as mass lesions with high and equal density mixed areas and well-defined boundaries. The lesions were poorly-enhanced heterogeneously following contrast enhancement, and speckled calcification and bony erosion was shown clearly on bone-window scans. Upon MRI, the lesions were shown as a hypointensity on T1-weighted scans and a mixed hyper- and hypointensity on T2-weighted scans. In certain cases, the tumors pressed or encased the internal carotid artery (ICA), and also thinned and occluded the ICA entering the cavernous sinus. In addition, the lesions displaced the surrounding brain tissues, but no cerebral edemas were observed.

### Pathological diagnoses

Histological analyses were performed in all cases, and diagnoses of chondromas were confirmed in 14 patients and chondrosarcomas in five. Additionally, immunohistochemical analyses were performed in seven cases, and the tumors were negative for epithelial membrane antigen (EMA) and cytokeratin (CK) and positive for S100.

### Follow-up

A total of 15 patients were followed up for a mean interval of 67.2 months (range, 5–140 months). Two patients experienced recurrence and have been monitored up to the present day. A complete or partial recovery was recorded in four patients with oculomotor nerve palsies, two with poor vision, six with abducens palsy, three with facial and acoustic nerve disorders and two with hoarseness. No patients underwent radiotherapy. Following surgery, the average KPS was 78.7. Among the 17 patients who were followed up, 13 (76.5%) had a normal life (KPS, 80–90) in society, three (17.6%) had moderate disabilities (KPS, 60–70) and one (5.9%) had severe disabilities (KPS, 50).

## Discussion

Chondrosarcomas and chondromas of the cranial base are rare tumors whose derivation is indicated to be from the remnants of embryonic cartilage tissue in the cartilaginous junction of cranial base sutures, or from chondrocytes in the areas of residual enchondral cartilage (1,4–7). In the present study, the mean age of occurrence ranged from the third to fourth decade of life, with no gender preference observed. Cranial base chondromas and chondrosarcomas usually arise in the central area of ossification or the suture, including the sphenopetrosal, sphenoclival or petroclival junctions, and most frequently are located in the parasellar area of the middle cranial base, extradurally. Additionally, these tumors frequently invade the cavernous sinus and extend into the posterior cranial fossa. The lesions develop and invade cranial nerves and major blood vessels gradually due to the slow growth of the tumors, thus accounting for the long duration of the symptoms. The most frequent clinical presentations are deficits of the cranial nerves, including the abducent, optic, acoustic-facial and lower cranial nerves, and chronically increased intracranial pressures and epilepsies (8,9).

Chondromas are recognized as a dyschondroplasia caused by developmental errors in enchondral ossification. Notably, Ollier’s disease and Maffucci’s syndrome are also characterized by dyschondroplasia. Ollier’s disease is referred to as a multiple enchondromatosis without dysplasia of other tissues or organs (10,11). Maffucci’s syndrome is a non-hereditary mesoblastic dysplasia and is characterized by the presence of multiple enchondromas and cutaneous and/or other parenchymatous hemangiomas (12–15). Although Ollier’s disease or Maffucci’s syndrome associated with intracranial neoplasms are extremely rare, cranial base chondromas or chondrosarcomas are believed to be a partial representation of the two diseases (10,14–16). In the present study, two patients were diagnosed with Maffucci’s syndrome associated with a cranial base chondrosarcoma.

Using neuroradiology, intracranial chondromas or chondrosarcomas are commonly imaged as a mass lesion located in the extradural lateral-middle areas of the cranial base, and particularly present as a single lesion in the parasellar region (1). The lesion can extend from the middle cranial fossa to the posterior cranial fossa, and can be imaged as a dumbbell profile. The lesion invading the jugular foramen can result in the widening of this foramen (5). As presented in the current study, the tumors normally arise in skeletal structures and are frequently accompanied by calcification and ossification. CT scans can reveal these characteristics well, and thus may be more useful than MRI for the presumptive diagnoses. By contrast, compared with CT, MRI is better for revealing the associations between lesions and surrounding anatomical structures, particularly those involving the ICA. According to the presenting symptoms and signs, and the neuroradiological examinations, a pre-operative diagnosis of chondroma or chondrosarcoma can be presumed.

Chondrosarcomas and chondromas can and should be distinguished from chordomas (17,18). Chordomas arise from the remnants of the primitive notochord and are commonly located in the middle line of the clivus, where the bone may be observably eroded and even completely replaced by the tumor tissues. Chondrosarcoma and chondromas are occasionally difficult to differentiate from chordomas morphologically. In such cases, an immunohistochemical analysis is necessary, with positive staining for CK, EMA and S-100 protein indicating a diagnosis of chordoma (15–18). The tumors from the patients of the present study all tested negative in the immunohistochemical analysis, and thus none were chordomas.

The treatment of cranial base chondromas and chondrosarcomas is a formidable challenge. Radiotherapy for these tumors remains controversial (19–21), but several studies have shown that proton-beam radiation achieves high rates of local tumor control and survival following maximal surgical resection (22–25). Surgical resection still plays a major role in the treatment for these tumors, but due to the invasion into cranial nerves and critical vascular structures, the goal of total tumor resection is difficult to achieve. The most effective surgery must remove the tumor tissue completely and avoid the incidence of complications overall (26–31). In the present study, this latter strategy was undertaken, resulting in no mortalities, a mean survival time similar to other studies and excellent follow-up outcomes.

The selection of surgical approaches to the tumors are discussed in certain studies (29,31). In order to plan the surgical approaches more accurately, the chondromas and chondrosarcomas of the cranial base are classified as the parasellar, straddle and posterior cranial fossa types, separating types by the location and growth direction of tumors. Patients with the parasellar type usually have deficits of cranial nerves III to VI, the most common being a sixth nerve palsy. The frontotemporal or preauricular subtemporal-infratemporal approach should be applicable to these patients. Tumors of the straddle type extend from the middle fossa to the posterior cranial fossa. Patients with this type have deficits of cranial nerves III to VIII. The tempo-occipital transtentorial or presigmoid supratentorial-infratentorial approach may be applicable to the patients with this type. The suboccipital far-lateral approach or the retrosigmoid approach can be used for the patients with the posterior cranial fossa type, who present with deficits of cranial nerves VII, VIII and the lower cranial nerves. The selection of suitable approaches is extremely significant in the direct visualization of tumors. Furthermore, skilled microsurgical techniques and good surgical judgment learned from experience are also essential to achieve a complete resection (29,31). These techniques and methods, as applied to the patients in the present study, likely contributed to the positive outcomes reported.

Chondromas and chondrosarcomas of the cranial base are deeply located and surrounded by significant complex neurovascular structures, thus surgical removal is challenging. In the majority of cranial base chondromas or chondrosarcomas, the pre-operative diagnosis can be presumed on the basis of neuroradiological examinations and presenting symptoms and signs. Occasionally it is difficult to differentiate chondrosarcomas/chondromas from chordomas, and immunohistochemistry is useful for the this purpose. Surgical resection is the principal treatment for cranial base chondromas or chondrosarcomas; this can prolong survival rates, but the surgical approach must be carefully planned for the individual tumor type and location.

## Abbreviations

CT: computed tomography
MRI: magnetic resonance imaging
ICA: internal carotid artery
KPS: Karnofsky performance score
